# Emotional anticipation for dynamic emotional faces is not modulated by schizotypal traits: A Representational Momentum study

**DOI:** 10.1177/17470218241253703

**Published:** 2024-05-16

**Authors:** Joana Grave, Sara Cordeiro, Nuno de Sá Teixeira, Sebastian Korb, Sandra Cristina Soares

**Affiliations:** 1 William James Center for Research (WJCR-Aveiro), Department of Education and Psychology, University of Aveiro, Aveiro, Portugal; 2 Center for Health Technology and Services Research (CINTESIS@RISE), Department of Education and Psychology, University of Aveiro, Aveiro, Portugal; 3 Department of Education and Psychology, University of Aveiro, Aveiro, Portugal; 4 Department of Psychology, University of Essex, Colchester, UK; 5 Department of Cognition, Emotion, and Methods in Psychology, University of Vienna, Vienna, Austria

**Keywords:** Schizotypal traits, schizophrenia, emotion processing, dynamic facial expression, Representational Momentum, social cognition

## Abstract

Schizotypy, a personality structure that resembles schizophrenia symptoms, is often associated with abnormal facial emotion perception. Based on the prevailing sense of threat in psychotic experiences, and the immediate perceptual history of seeing others’ facial expressions, individuals with high schizotypal traits may exhibit a heightened tendency to anticipate anger. To test this, we used insights from Representational Momentum (RM), a perceptual phenomenon in which the endpoint of a dynamic event is systematically displaced forward, into the immediate future. Angry-to-ambiguous and happy-to-ambiguous avatar faces were presented, each followed by a probe with the same (ambiguous) expression as the endpoint, or one slightly changed to express greater happiness/anger. Participants judged if the probe was “equal” to the endpoint and rated how confident they were. The sample was divided into high (*N* = 46) and low (*N* = 49) schizotypal traits using the Schizotypal Personality Questionnaire (SPQ). First, a forward bias was found in happy-to-ambiguous faces, suggesting emotional anticipation solely for dynamic faces changing towards a potential threat (anger). This may reflect an adaptative mechanism, as it is safer to anticipate any hostility from a conspecific than the opposite. Second, contrary to our hypothesis, high schizotypal traits did not heighten RM for happy-to-ambiguous faces, nor did they lead to overconfidence in biased judgements. This may suggest a typical pattern of emotional anticipation in non-clinical schizotypy, but caution is needed due to the use of self-report questionnaires, university students, and a modest sample size. Future studies should also investigate if the same holds for clinical manifestations of schizophrenia.

## Introduction

### Schizophrenia and emotion perception

Schizophrenia is a severe psychiatric disorder, marked by a complex pattern of disorganised (e.g., speech), positive (e.g., hallucinations, delusions), and negative symptoms (e.g., avolition, anhedonia) (American Psychiatric Association, 2013). In addition, schizophrenia is characterised by deficits in social cognition (M. F. Green et al., 2015). These are correlated with poor functioning outcomes (Halverson et al., 2019), present at the early stages of the disorder (Healey et al., 2016), and resistant to antipsychotics (e.g., Kucharska-Pietura & Mortimer, 2013).

One of the most affected social cognitive abilities is emotion perception (M. F. Green et al., 2015). Overall, individuals with schizophrenia show large and consistent deficits in the recognition and discrimination of emotional faces (see Kohler et al., 2010; Savla et al., 2013). For instance, individuals with schizophrenia tend to misattribute anger or fear to neutral faces (e.g., Habel et al., 2010; Kohler et al., 2003; Pinkham et al., 2011; Premkumar et al., 2008). This aligns with a predominant sense of threat experienced in psychosis (Underwood et al., 2016), especially in social contexts (e.g., Veling et al., 2016). Because being able to perceive others’ emotional states is critical for successful emotional communication, an aberrant perception of emotional faces can negatively affect patients’ social behaviour, thus contributing to a cascade of socio-emotional difficulties (e.g., Hooker & Park, 2002). Yet, individuals with schizophrenia (compared with healthy controls) seem to be overconfident in their ability to accurately identify others’ emotional (e.g., Köther et al., 2018; Perez et al., 2020) and mental states (e.g., Köther et al., 2012). This overconfidence, which extends to lower-level visual perception as well (e.g., Moritz et al., 2014), is believed to play a role in the development and maintenance of positive symptoms, such as delusions (Balzan, 2016). Furthermore, deficits in emotion perception might be a potential endophenotype of schizophrenia, as they are also observed (although less severely) in unaffected first-degree relatives (Allott et al., 2015), clinical high-risk (Allott et al., 2014) and, of foremost importance to this study, schizotypy (see Giakoumaki, 2016; Zouraraki et al., 2023).

### Schizotypy and emotion perception

Schizotypy was first described as a manifestation of genetic risk of schizophrenia (e.g., Meehl, 1989) and is currently defined as a personality structure characterised by psychological factors that resemble the positive, negative, and disorganised symptoms of schizophrenia (Ettinger et al., 2014; Nelson et al., 2013). In addition, schizotypy and schizophrenia seem to overlap on some genetic, cognitive, neurobiological, and psychosocial levels, thus suggesting a psychotic continuum, ranging from low schizotypal traits in the general population to the clinical manifestation of schizophrenia (occurring in some extreme cases of schizotypy, and/or under adverse environmental factors) (Nelson et al., 2013). Indeed, although the presence of schizotypal traits is not enough for a schizophrenia diagnosis, individuals with high schizotypal traits have an increased risk of developing a psychotic disorder (Debbané et al., 2015). Hence, the study of schizotypal traits in the general population has gained increased attention, as it can provide critical knowledge about the mechanisms underlying the clinical manifestation of schizophrenia while eliminating clinical confounders, namely medication side-effects, comorbidities, chronic hospitalisation, and psychomotor slowing (Barrantes-Vidal et al., 2015; Ettinger et al., 2014).

Concerning emotion perception, there is evidence that higher schizotypal traits are associated with abnormal facial emotion perception (e.g., Brown & Cohen, 2010; Dawes et al., 2021; Fusar-Poli et al., 2022; Morrison et al., 2013; Yan et al., 2020). For instance, using a visual search paradigm, Uono et al. (2021) found that schizotypal traits—evaluated with the Schizotypal Personality Questionnaire (SPQ; Raine, 1991)—were negatively correlated with the effectiveness for detecting normal versus “anti-facial” expressions^1^ of happiness/anger among a crowd of neutral faces. The authors proposed that individuals with high schizotypal traits failed to detect emotional faces in a privileged manner because they overattribute (emotional) salience to non-salient stimuli (Uono et al., 2021). This is consistent with a positive correlation between schizotypal traits and the activation of the right posterior superior temporal sulcus during neutral face perception (Yan et al., 2020). In this vein, it is possible that the aberrant attribution of salience described in neurobiological models of psychosis—presumably caused by elevated dopaminergic signalling (Howes & Nour, 2016; Kapur, 2003)—represents a schizophrenia vulnerability that may manifest at a subclinical level as well (e.g., van Os et al., 2017).

### Dynamic emotional faces

Despite the constant dynamic changes of faces in the real world, which convey dynamic cues of verbal and non-verbal communication, such as gaze, and emotional state (Dobs et al., 2018; Krumhuber et al., 2013), the exploration of how individuals with schizotypal traits perceive such stimuli remains largely unexplored (see Zouraraki et al., 2023). Remarkably, studies have shown that observers can anticipate others’ emotional states based on subtle changes in facial expression (Jellema et al., 2011; Marian & Shimamura, 2013; Palumbo & Jellema, 2013) and that this ability is altered in neurodevelopmental disorders (Palumbo et al., 2015, 2018). For instance, a series of studies demonstrated that neutral faces initially displaying a happy (happy-to-neutral) or angry (angry-to-neutral) expression are rated as more angry or happy, respectively, which indicates a bias towards the anticipated emotional state (e.g., Jellema et al., 2011; Palumbo et al., 2015, 2018; Palumbo & Jellema, 2013). According to the authors, this phenomenon reflects mechanisms of emotional anticipation triggered by the immediate perceptual history of others’ facial expressions, but they could also be explained by Representational Momentum (RM).

### Representational Momentum

RM is a perceptual phenomenon where the final location of a moving target is systematically shifted forward, extending into the immediate future and aligning with the direction of motion (e.g., Freyd & Finke, 1984; see Hubbard, 2010, 2014). Despite the lack of consensus on the mechanisms underlying RM, this phenomenon is believed to compensate for delays in sensory and motor systems (Hogendoorn, 2020), and to be influenced by the characteristics of the observer, such as expertise and psychopathology (see Hubbard, 2014 for a theoretical review). Yet, only a few studies have looked at whether RM effects vary depending on traits along the psychotic continuum (e.g., De Sá Teixeira et al., 2013; Jarrett et al., 2002; Tulver et al., 2019; Vettise, 2012). For instance, Jarrett et al. (2002) observed a non-significant trend towards a larger RM effect in individuals with schizophrenia and those with high schizotypal traits (top 16% of scorers on the SPQ), compared with individuals with low schizotypal traits (bottom 16% of scorers on the SPQ). This may stem from a failure to inhibit the automatic process of motion extrapolation in schizophrenia and schizotypy. Nevertheless, it is important to note that this effect was just shy of the statistical threshold, likely influenced by an underpowered sample size. Using a non-clinical sample, Vettise (2012) later found a positive correlation between RM for implied motion and the SPQ disorganised factor, but not the cognitive-perceptual SPQ factor (which does not support the author’s main hypothesis). In contrast, a multi-paradigm study conducted by Tulver et al. (2019) revealed that individuals scoring higher on positive schizotypy (assessed with the cognitive-perceptual SPQ-Brief factor) exhibited a reduced reliance on their prior knowledge in visual perception. This was evidenced by, for instance, a smaller RM for smooth motion. Given the differences across these studies in terms of response modality (e.g., probe judgement versus spatial adjustment), motion (e.g., implied versus smooth), and sample characterisation (e.g., non-clinical versus clinical population), direct comparisons between them present significant challenges. In addition, the exclusive use of non-emotional stimuli (e.g., jumping man) in these studies raises doubts about whether schizotypal traits influence RM in response to changes in emotional stimuli.

The RM effect has been observed not only for basic stimuli but also for socially relevant events, including pain (Dozolme et al., 2018; Prigent et al., 2018), social threat (Greenstein et al., 2016), gaze (Hudson et al., 2009; Hudson & Jellema, 2011), and emotional faces (e.g., Courgeon et al., 2010; Senior et al., 2020; Uono et al., 2014; Yoshikawa & Sato, 2008, but see Thornton, 2014 for conflicting findings). For instance, Greenstein et al. (2016) found that the final location of a ring moving towards a stationary dot was more strongly displaced forward when both stimuli were preceded by a threatening vignette (e.g., the ring was an assailant holding a weapon and approaching the victim, represented by the dot) than a neutral vignette (e.g., the ring was a person holding a sandwich). This suggests that RM is greater for threatening social events, which may reflect an adaptative anticipation phenomenon (e.g., Gladwin & Vink, 2020). Yoshikawa and Sato (2008) conducted a study where participants were shown neutral-to-emotional human faces, each followed by a static probe. Participants were then asked to adjust the probe’s facial expression to match the final frame of the animation. Probes were systematically adjusted to a more intense version, regardless of the emotional category. A similar effect was reported by Courgeon et al. (2010), but only for emotional faces ending at medium intensity (50%), with higher intensities (70% and 90%) resulting in a backward bias—an effect opposite to RM. They also observed that the magnitude of this effect depended on the emotion type, with anger, disgust, fear, and surprise producing an “Emotional Momentum” (Courgeon et al., 2010).

To our knowledge, the RM effect for emotional faces has not yet been investigated in schizophrenia. Regarding schizotypal traits, Uono et al. (2015) found that scores on the SPQ were positively correlated with a tendency to exaggerate the intensity of the final frame of neutral-to-happy and neutral-to-fearful faces (at 52%, 80%, or 108% intensity), regardless of the emotional category (Uono et al., 2015). However, this effect extended to static faces as well, thus pointing to a broader pattern of aberrant emotion perception in schizotypal traits, rather than an exaggerated RM for socio-emotional events. Considering that RM is greater for emotional faces ending at a lower intensity (Courgeon et al., 2010), it is plausible that presenting emotional faces changing towards neutral/ambiguous expressions amplifies a possible difference between “high” and “low” schizotypy—especially because the perception of neutral/ambiguous expressions is highly dependent on contextual cues (see Wieser & Brosch, 2012).

### Current study

**Figure 1.**
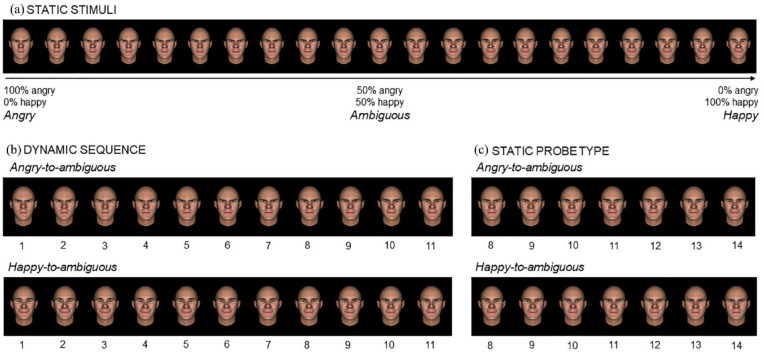
Example of the 21 static facial expressions of a male avatar (Panel a) used to create the angry-to-ambiguous and happy-to-ambiguous animations (Panel b) and the corresponding probes (Panel c).

In this preregistered study, we aimed to investigate the effects of schizotypal traits in emotional anticipation for dynamic emotional faces, using the RM framework. For that, we created a probe judgement task with ambiguous faces (depicting 50% of happiness and 50% of anger) of male and female avatars that began with a fully angry (henceforth “angry-to-ambiguous”) or fully happy expression (henceforth “happy-to-ambiguous”). Compared with earlier studies with neutral faces as endpoints (Jellema et al., 2011; Marian & Shimamura, 2013; Palumbo & Jellema, 2013), the use of ambiguous faces provided a more ecologically valid transition between anger and happiness, as “real” faces typically portray features of multiple emotions rather than a single or “blank” expression (Aviezer et al., 2008; Hassin et al., 2013). After each animation, a probe of the same avatar was exhibited, depicting either the same expression (i.e., ambiguous) or one slightly displaced towards a more angry/happy expression by one, two, or three frames (Figure 1). In each trial, participants were asked to make equal/different judgements (first-order task) and to rate how confident they felt of their response (second-order task). The second-order task was added to provide a quantitative measure of metacognition, related to the sense of being correct about one’s perceptions, memories, and decisions (see Fleming et al., 2012; Heyes et al., 2020). A forward bias (consistent with RM) occurred when probes depicting an emotion intensity beyond the final frame, in the direction of the anticipated motion (e.g., slightly angrier probes in happy-to-ambiguous conditions), were incorrectly judged as “equal”. Conversely, a backward bias (opposite to RM) occurred when probes showing a previous emotional state (i.e., slightly happier probes in happy-to-ambiguous conditions) were incorrectly judged as “equal”.

Schizotypal traits were assessed with the SPQ. The participant sample was divided into high and low SPQ groups by a median split of the total questionnaire score (e.g., Ahn et al., 2020). With respect to the first-order task, we expected a main effect of emotion, with a greater RM effect for happy-to-ambiguous than angry-to-ambiguous faces. Because happy-to-ambiguous faces display gradual increases in anger (and decreases in happiness), this would be consistent with a larger forward bias for potentially threatening events, as suggested by prior research (Greenstein et al., 2016). Moreover, we anticipated a significant SPQ Group by Emotion interaction. Specifically, we hypothesised that the high (versus low) SPQ group would demonstrate larger RM for happy-to-ambiguous faces, thus supporting previous reports of an enhanced threat anticipation in schizophrenia (e.g., Kvarta et al., 2021) and psychotic proneness (e.g., Paetzold et al., 2023; Reininghaus et al., 2016). Considering the evidence indicating overconfidence in emotion perception errors in schizophrenia (e.g., Köther et al., 2018; Perez et al., 2020) and cognitive domains at a subclinical level, such as delusional ideation (see Hoven et al., 2019), we also anticipated a main effect of SPQ group in the second-order task, thus revealing a tendency towards overconfidence in biased perceptual judgements in the high SPQ group. Finally, we also expected a significant SPQ Group by Emotion interaction in the second-order task such that group difference would be greater for happy-to-ambiguous faces (compared with angry-to-ambiguous), suggesting greater or specific overconfidence in threat appraisals.

Finally, we implemented an ambiguity threshold task, which involved categorising the emotion portrayed by the male and female avatars using a two-alternative forced-choice staircase method. Unlike the main task, each stimulus was presented statically, representing a specific point along the full spectrum of anger/happiness intensity. This enabled the assessment of the ambiguity threshold for each participant and avatar face, thus accounting for individual variation. Hypotheses, sample size, study design, and analysis plan are preregistered at Open Science Framework (OSF) and can be accessed at https://osf.io/v9kwz/.

## Methods

### Sample size justification

Sample size was calculated using a simulated power analysis with the ANOVA_Power Shiny App (Lakens & Caldwell, 2021), with 2,000 simulations and an alpha-level of .05. We defined a mixed 2 × 2 design with Group (high SPQ, low SPQ) as between-subjects factor and Emotion (happy-to-ambiguous, angry-to-ambiguous) as within-subjects factor. We were mainly interested in the Group by Emotion interaction. Assuming the high SPQ group would have a mean of 0.45 for happy-to-ambiguous and −0.1 for angry-to-ambiguous trials, whereas the low SPQ group would have a mean of 0.2 for happy-to-ambiguous and 0.1 for angry-to-ambiguous trials,^2^ we estimated that 52 participants per group (*N* *=* 104) are necessary to obtain 81% power for the three-way interaction.

### Participants

Participants were recruited from the University campus through social media platforms, flyers, and mailing lists. Participants were all Portuguese speakers, aged between 18 and 40 years, and with normal or corrected-to-normal vision. Exclusion criteria were (a) failure to understand instructions and procedures; (b) self-report of medical or neurological disorders that may affect brain function (e.g., history of seizures, head trauma with unconsciousness for >15 min); (c) formal diagnosis of mental disorders; (d) formal diagnosis of psychotic disorders in biological first-degree relatives; (e) and current psychotropic medication intake.

A total of 114 participants (81 women; 71.05%) were recruited. Of those, five were excluded for having a formal diagnosis of mental disorder, three for not completing the questionnaires, one for not understanding the instructions, and one for computer errors. The sample included 104 participants (73 women; 70.19%), aged between 17 and 39 (*M* *=* 21.48; *SD* *=* 4.18). All procedures were approved by the University Ethics Committee (03-CED/2020) and conducted in accordance with the guidelines of the Declaration of Helsinki and the American Psychological Association. Participants received either course credits (Psychology students) or a stamp for a 5€ voucher in return for participation.

### Measures

Groups were divided based on the total score of the Portuguese version of the SPQ (Paixão & Quelhas, 2013), a self-report questionnaire to screen the general population for Schizotypal Personality Disorder (Raine, 1991). The SPQ contains 74 items with dichotomous response format (yes/no), distributed across nine dimensions that can be merged into three factors based on the three-factor model of schizotypy (Raine et al., 1994): cognitive-perceptual (ideas of reference, odd beliefs or magical thinking, unusual perceptual experiences, suspiciousness), interpersonal (social anxiety, lack of friends, constricted affect, suspiciousness), and disorganised (odd or eccentric behaviour, odd speech). The factor scores, dimension scores, and total score are derived by summing the “yes” responses across all items. Total score range from 0 to 74, with higher scores indicating higher schizotypal traits. Our sample showed acceptable levels of internal consistency, with a Cronbach’s alpha of .90 for the total score and between .63 and .81 for the nine dimensions.

For a more comprehensive assessment of psychotic proneness, we also included measures of psychotic-like experiences that may manifest at a subclinical level. For that, we used the Portuguese version of the Launay-Slade Hallucination Scale-Revised (LSHS-R; Castiajo & Pinheiro, 2017) to evaluate different forms of hallucinations, and the Portuguese version of the Peters et al. Delusional Inventory (PDI-21; Pimentel et al., 2017) to evaluate the multi-dimensionality of delusional ideation. In addition, we used the Portuguese version of the State-Trait Inventory for Cognitive and Somatic Anxiety (STICSA; Barros et al., 2022) to statistically control for the effects of state anxiety (STICSA-1) and trait anxiety (STICSA-2).

Finally, a brief cognitive assessment was performed using the Portuguese version of the Trail Making Test Part A (TMT-A; Cavaco et al., 2013) and the Letter-Number Span (Wechsler, 2008). See Supplementary materials for a more detailed description of the measures.

### Stimuli

A particular strength of this study lies on the use of avatar stimuli, which enabled precise control over low-level features (e.g., symmetry of morphology and expression, alignment of eyes and most face elements, closed mouth) and emotional changes in facial expression. Two male and two female avatar faces (front view) with neutral expressions were created with FaceGen Modeller 3.5.3 (Singular Inversions Inc.) using the following setup: (a) European race, (b) 30 years of age, (c) average shape and texture on the caricature scale, (d) full symmetry, and (e) gender shape to “very male” or “very female”. Avatar faces were saved and imported into FacsGen (Krumhuber et al., 2012).

For each avatar, a 100% angry expression (AU4 Brow lowerer = 100%, AU5 Upper lid raiser = 100%, AU7 Lids tightener = 50%, AU9 Nose wrinkle = 30%, AU23 Lip tightener = 100%) and a 100% happy expression (AU6 Cheek raiser = 100%, AU12 Lip corner puller = 100%) were created (all with a closed mouth), as well as 19 gradual transitions between them (Figure 1a). This resulted in 21 pictures per avatar (84 stimuli in total). The intermediate stimulus corresponded to an ambiguous expression, with 50% anger and 50% happiness. All emotion changes were made based on the Facial Action Coding System (FACS; Ekman et al., 2002) by a certified FACS coder using FacsGen. For similar procedures, see Korb et al. (2023).

### Probe judgement task

**Figure 2.**
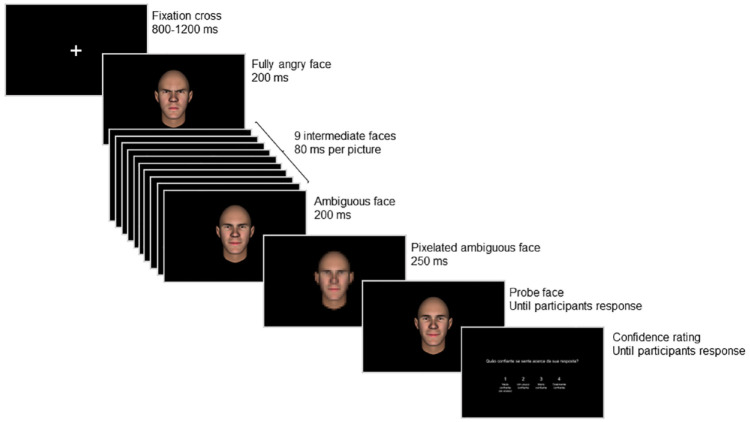
Representation of a trial of the probe judgement task.

Experimental tasks were programmed with PsychoPy3 (Peirce et al., 2019). Each trial started with a white fixation cross, for a random duration of 800 to 1200 ms, followed by a sequence of avatar faces, gradually changing from a fully angry to an ambiguous expression (angry-to-ambiguous) or from a fully happy to an ambiguous expression (happy-to-ambiguous). Each animation contained 11 pictures (henceforth “frames”), all belonging to the same avatar (Figure 1b). The first frame (i.e., fully angry or happy expression) was shown for 200 ms, followed by nine intermediate frames presented for 80 ms each, and the final frame for 200 ms (Figure 2). Importantly, the final frame always displayed an ambiguous expression, irrespective of the emotion condition. Following each animation, a mask (a pixelated picture of the final frame) was presented for 250 ms, succeeded by a probe that remained visible until participants’ response (for similar procedures, see Dozolme et al., 2018; Hudson et al., 2016; Prigent et al., 2018). The probe was a static face of the same avatar, depicting the same expression as the final frame (i.e., ambiguous) or one slightly displaced towards an angrier or happier expression by one, two, or three frames, thus producing seven levels per animation (Figure 1c).

All stimuli were presented at a 13° visual angle, corresponding to the perceived dimension of a human face at about 100 cm distance—the average personal space during social interactions with an unfamiliar person (Hecht et al., 2019). Participants were required to judge if the facial expression depicted by the probe was “equal to” or “different from” the final frame of the animation, by pressing the “up” or “down” keys on the keyboard, respectively. No feedback was provided. Immediately after response, participants indicated, from 1 (“*not confident*”) to 4 (“*very confident*”), how confident they were in their response, by pressing the “1”, “2”, “3”, or “4” keys on the keyboard (Figure 2).

The probe judgement task contained 168 trials (2 Emotion × 2 Avatar Sex × 2 Avatar Identity × 7 Probe × 3 repetitions) and was preceded by five training trials, all in a randomised order. None of the stimuli used in the training trials were shown in the main task.

### Ambiguity threshold task

Participants completed an interleaved staircase task to estimate the ambiguity threshold for each participant and avatar, using static stimuli. In each trial, a white fixation cross was shown at the centre of the screen, for a random duration of 800 to 1,200 ms, followed by one of the static pictures used to create the animations of the probe judgement task. All stimuli were presented at a 13° visual angle. Participants indicated whether the avatar face depicted anger or happiness, by pressing the “l” or “a” key on the keyboard, respectively. No feedback was provided. Emotion intensity (21 pictures per avatar) gradually changed towards greater anger or happiness based on the last response in each stair, following a two-up, two-down rule: after two successive angry or happy responses, emotion intensity changed towards a fully angry or happy expression. Two randomly interleaved stairs per avatar were used, one starting with a fully angry and one with a fully happy expression. Furthermore, the size of intensity steps each time a stair was presented was three pictures before the first reversal and one picture afterwards. Each stair ended after a minimum of four reversals (constrained to a minimum of five presentations per stair).

### Procedures

After signing the consent form and completing the STICSA-1 (in paper), participants were seated upright, as comfortably as possible, in front of an LG 24GL650-B monitor (1920 × 1080 pixels resolution; 100 Hz refresh rate), with its centre located 60 cm from participants’ forehead. They were asked to gaze at the fixation cross before the stimulus presentation. Participants performed the probe judgement task, followed by the ambiguity threshold task. Tasks were run in PsychoPy3 and lasted about 20 and 10 min, respectively. Response time (ms) and response key were recorded in both tasks. Next, participants completed the socio-demographic questionnaire, the SPQ, LSHS, PDI, and STICSA-2 (in the platform https://forms.ua.pt/), as well as the TMT-A and Letter-Number Span (in paper). Finally, participants were fully debriefed about the purposes of the study and were rewarded for their participation.

### Statistical analyses

#### Preregistered statistical analyses

Data and code are publicly available at OSF and can be accessed at https://osf.io/apjcn/. Trials with response times shorter than 250 ms in the first-order task were considered outliers and excluded from analyses (*N* *=* 29; 0.16%). Data were pre-processed in *R* (Version 4.2.1; R Core Team, 2022) using RStudio (RStudio Team, 2022), results were saved as .xlsx using the *writexl* package (Ooms, 2021), and analysed in jamovi (Version 2.3; The Jamovi Project, 2022). We used the *ggpubr* package (Kassambara, 2020) for data visualisation.

Concerning the first-order task, the main analysis consisted of a 2 × 2 repeated-measures analysis of variance (ANOVA) with SPQ Group (between-subjects factor) and Emotion (within-subjects factor). Greenhouse-Geisser correction was applied in case of violation of sphericity, assessed via Mauchly’s test. Post hoc analyses with Tukey correction for multiple comparisons were computed, and the alpha level was set to .05. Dependent variables were Point of Subjective Equality (PSE) and Just Noticeable Difference (JND), both estimated by fitting a logistic probability density function to the proportion of “equal” responses (with three free parameters: distribution mean, which provides the PSE estimate, distribution deviation, which allows for an estimation of JND, and a scaling factor; for a similar procedure, see Dozolme et al., 2018).^3^ The PSE provided an estimation for the probe level for which participants were more likely to respond “equal”. Thus, a PSE equal to 11 (corresponding to the animation endpoint) suggests an accurate mnesic representation of the final frame. In contrast, a PSE larger than 11 suggests a forward bias, whereas a PSE smaller than 11 has a backward bias.

Given the reported implicit effect of face sex on emotion perception (Becker et al., 2007; Hess et al., 2009; Korb et al., 2023) and women’s advantage in recognising emotional faces (e.g., Bek et al., 2022), two exploratory repeated-measures ANOVAs were conducted to test the effects of sex. Those were composed by Emotion and Avatar Sex (within-subjects factor) or Participant Sex (between-subjects factor). Should any effects of sex emerge, the respective variable would be included in the analyses. We also controlled for the effects of total SPQ, disorganised SPQ, cognitive-perceptual SPQ, and interpersonal SPQ factors, using these variables individually as covariates in repeated-measures ANOVAs with Emotion. Total PDI and LSHS (to control for the effects of psychotic-like experiences), total STICSA-1 and STICSA-2 (to control for the effects of state anxiety and trait anxiety, respectively), and TMT-A and Letter-Number Span (to control for the effects of cognition) were also individually added as covariates. All covariates were mean-centred.

For the second-order task, we computed the mean of the product of the proportion of “equal” errors and the confidence mean of “equal” errors.^4^ Only “equal” errors were used as we aimed to investigate subjective confidence in the context of a perceptual bias. The main analysis consisted of a repeated-measures ANOVA with Emotion and SPQ Group. The effects of total SPQ, total PDI, total LSHS, total STICSA-1 and STICSA-2, TMT-A, and Letter-Number Span were also controlled for, using these variables individually as covariates, and two exploratory analyses with Avatar Sex and Participant Sex were performed (both having Emotion as a within-subjects factor).

#### Non-preregistered analyses

Exploratory repeated-measures ANOVAs with distinct groups were computed for both the first- and second-order task: “high” and “low” based on PDI and LSHS median split, to further explore the effect of psychotic-like experiences, and “high” and “low” based on SPQ tertiles, discarding the group with “medium” SPQ scores (for similar procedures, see Laycock et al., 2019). Spearman’s rank correlation was used to investigate whether PSE and subjective confidence correlated with total SPQ, total PDI, and total LSHS, as well as disorganised, cognitive-perceptual, and interpersonal SPQ factors.

Concerning the first-order task, we also ran a repeated-measures ANOVA to test the effects of Probe Level and Emotion on the proportion of “equal” responses, serving as a validation check. Moreover, a one-tailed one-sample *t-*test was performed to explore whether PSE was significantly larger than 11 (corresponding to the animation endpoint).

Concerning the ambiguity threshold task, the ambiguity threshold was calculated as the mean of the picture level where reversals (with a size intensity set of 1) took place. Values below 11 suggest that faces with angrier than happier cues were perceived as being ambiguous. First, a two-tailed one-sample *t*-test was performed to explore whether the ambiguity threshold was significantly different from 11 (the intermediate level of emotion change). Second, a repeated-measures ANOVA with Avatar Sex and SPQ Group was conducted. The ambiguity threshold was also correlated with total SPQ, total PDI, and total LSHS, as well as disorganised, cognitive-perceptual, and interpersonal SPQ factors. Finally, JND was computed and used as a dependent variable in a repeated-measures ANOVA with Avatar Sex and SPQ Group.

## Results

### Representational Momentum

Because exploratory analyses revealed no effect of Avatar Sex and Participant Sex on PSE, these variables were excluded from further analyses. Upon visual inspection, participants whose PSE for happy-to-ambiguous or angry-to-ambiguous faces fell below 5 or exceeded 16 (deviating three levels beyond the more extreme probes) were excluded (*N* *=* 9; 8.65%), as their distribution of “equal” responses, within the probed range, did not follow a consistent pattern. This step allowed a more reliable estimation of RM. The analysed sample thus comprised 95 participants (67 women; 70.53%), whose ages ranged from 18 to 39 years (*M* *=* 21.50; *SE* *=* 0.43). Of those, 46 participants were classified into the high SPQ group and 49 into the low SPQ group. Socio-demographic, psychological, and cognitive characteristics are described in Table S1 in the Supplementary materials.

**Figure 3.**
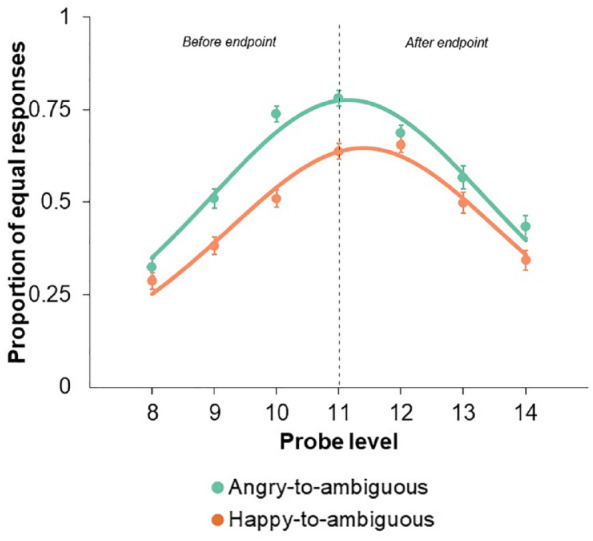
Proportion of “equal” responses as a function of Probe Level (data points), with Emotion as the curve parameter. The lines depict the best-fitting logistic probability density functions for each Emotion. Probe Level 11 corresponds to the animation endpoint, portraying a 50% happy and 50% angry expression. Probe Levels 8, 9, and 10 depict facial expressions occurring 3, 2, and 1 frames before the endpoint, respectively. Conversely, Probe Levels 12, 13, and 14 illustrate the anticipated continuation beyond the endpoint by 1, 2, and 3 frames, respectively. Error bars represent standard errors of the mean.

Concerning PSEs, we first found a significant main effect of Probe Level, *F*(6, 564) = 68.20, *p* *<* .001, η²*p* *=* .420, a significant main effect of Emotion, *F*(1, 94) = 44.71, *p* *<* .001, η²*p* *=* .322, and a significant Probe Level by Emotion interaction, *F*(6, 564) = 6.69, *p* *<* .001, η²*p* *=* .066. As the probe level approached 11 (representing the animation endpoint), the proportion of “equal” responses increased for angry-to-ambiguous faces (*M* = 0.78; *SE* = 0.02; 95% CI [0.74, 0.82]), whereas the peak proportion occurred at probe level 12 for happy-to-ambiguous faces (*M* = 0.66; *SE* = 0.02; 95% CI [0.61, 0.70]) (Figure 3).

**Figure 4.**
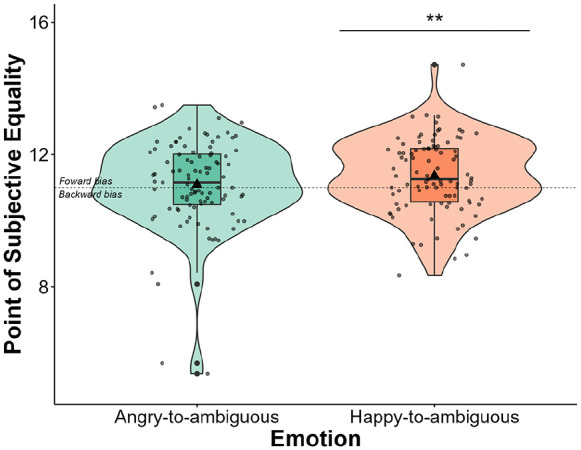
Violin plots with box plots displaying the distribution of PSEs across the angry-to-ambiguous and happy-to-ambiguous transitions. Violin plots represent the probability density of PSE, whereas box plots depict quartile distributions. Mean PSE values are indicated by a triangle symbol. Jittered points represent individual data points. Error bars represent standard errors of the mean. Results revealed that PSE was significantly larger than 11 (the animation endpoint) for happy-to-ambiguous faces (*p* = .001), but not for angry-to-ambiguous faces (*p* = .263). **p < .010.

A one-sample *t-*test revealed that PSE was significantly larger than 11 for happy-to-ambiguous faces (*M* *=* 11.36; *SE* *=* 0.11; 95% CI [11.13, 11.58]), *t*(94) = 3.11, *p* *=* .001, *d* *=* 0.319, 95% CI [0.112, 0.524], but not angry-to-ambiguous faces (*M* *=* 11.09; *SE* *=* 0.14; 95% CI [10.81, 11.36]), *t*(94) = 0.64, *p* *=* .263, *d* *=* 0.065, 95% CI [−0.136, 0.266] (Figure 4).

**Figure 5.**
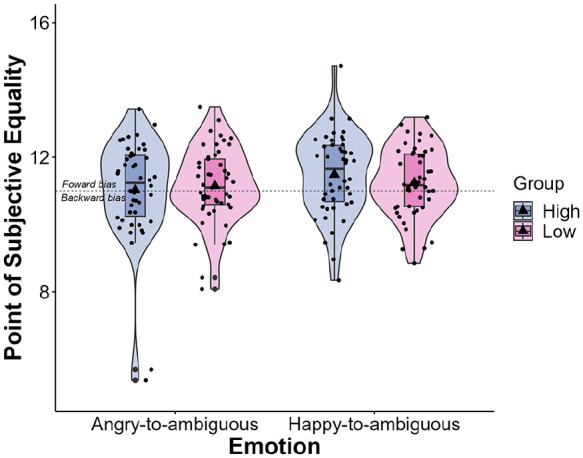
Violin plots with box plots displaying the distribution of PSE across the angry-to-ambiguous and happy-to-ambiguous transitions, categorised by SPQ Group. Violin plots represent the probability density of PSE, whereas box plots depict quartile distributions. Mean PSE values are indicated by a triangle symbol. Jittered points represent individual data points. Error bars represent standard errors of the mean.

Next, a repeated-measures ANOVA revealed a significant main effect of Emotion, *F*(1, 93) = 4.69, *p* *=* .033, η²*p* *=* .048. The PSE was significantly larger for happy-to-ambiguous faces than for angry-to-ambiguous faces. No main effect of SPQ Group, *F*(1, 93) = 0.06, *p* *=* .810, η²*p* *=* .001, and no significant SPQ Group by Emotion interaction were found, *F*(1, 93) = 2.18, *p* *=* .143, η²*p* *=* .023 (Figure 5).

PSEs were not affected by the covariates SPQ (total score, cognitive-perceptual factor, interpersonal factor, and disorganised factor), total LSHS, total STICSA-1, total STICSA-2, TMT-A, and Letter-Number Span (all *p* *<* .050)—the main effect of Emotion remained unchanged in these analyses. Interestingly, the covariate total PDI significantly interacted with Emotion, *F*(1, 93) = 4.42, *p* *=* .038, η²*p* *=* .045, which suggests that the main effect of Emotion on PSE varies depending on individuals’ scores on the PDI. To further explore this, the sample was divided into low (*N* *=* 48) and high (*N* *=* 47) groups based on the median split of PDI. Results showed a null PDI group effect, *F*(1, 93) = 0.182, *p* *=* .671, η²*p* *=* .002. The PDI Group by Emotion interaction approached but did not reach the significance threshold, *F*(1, 93) = 3.84, *p* *=* .053, η²*p* *=* .040. Particularly, the high PDI group exhibited a significantly larger PSE for happy-to-ambiguous stimuli (*M* = 11.43; *SE* = 0.16; 95% CI [11.11, 11.76]) than angry-to-ambiguous faces (*M* = 10.92; *SE* = 0.19; 95% CI [10.53, 11.30], *p* = .024). Conversely, the difference in PSE between happy-to-ambiguous (*M* *=* 11.28; *SE* *=* 0.16; 95% CI [10.96, 11.60]) and angry-to-ambiguous faces in the low PDI group was not statistically significant (*M* *=* 11.25; *SE* *=* 0.19; 95% CI [10.87, 11.64], *p* *=* .999).

To confirm whether the null effect of the SPQ Group was caused by the median split not providing enough granularity, the sample was also divided into two groups based on SPQ tertile: high SPQ (*N* *=* 30) and low SPQ (*N* *=* 35). Consistent with earlier findings, the SPQ Group by Emotion interaction failed to reach significance, *F*(1, 63) = 2.70, *p* *=* .105, η²*p* *=* .041. Furthermore, Spearman’s rank correlation showed no significant correlation between PSE and total SPQ (*r* = 0.129, *p* = .212), cognitive-perceptual SPQ (*r* = 0.097, *p* = .351), interpersonal SPQ (*r* = 0.077, *p* = .461), disorganised SPQ (*r* = 0.145, *p* = .160), total LSHS (*r* = 0.002, *p* = .984), and total PDI (*r* = −0.003, *p* = .976).

With respect to JND, descriptive analyses demonstrated that JND was greater for happy-to-ambiguous (*M* *=* 2.18; *SE* *=* 0.63; 95% CI [0.92, 3.44]) than angry-to-ambiguous faces (*M* *=* 1.60; *SE* *=* 0.08; 95% CI [1.45, 1.75]). However, this difference failed to reach the statistical significance criteria, with results indicating the absence of significant effects of Emotion, *F*(1, 93) = 0.78, *p* *=* .378, η²*p* *=* .008, SPQ Group, *F*(1, 93) = 0.928, *p* *=* .338, η²*p* *=* .010, and the SPQ Group by Emotion interaction, *F*(1, 93) = 0.983, *p* *=* .324, η²*p* *=* .010.

Finally, we tested the proportion of participants showing an RM greater than JND based on their SPQ group. This allowed us to explore whether individuals with high schizotypal traits demonstrated a tendency to anticipate emotions that exceeded their visual acuity for detecting changes in emotion intensity. Because data did not follow a normal distribution based on the Kolmogorov–Smirnov test, we conducted a Chi-square test. Results revealed no significant association between the proportion of participants with RM greater than JND and the SPQ group for both happy-to-ambiguous faces (nine of 46 participants in the high group; seven of 49 participants in the low group), χ²(1) = 0.47, *p* *=* .492, and angry-to-ambiguous faces (two of 46 participants in the high group; three of 49 participants in the low group), χ²(1) = 0.15, *p* *=* .699.

### Subjective confidence

Concerning weighted confidence, neither a main effect of SPQ Group, *F*(1, 93) = 0.10, *p* = .752, η²*p* = .001, nor a significant Group by Emotion interaction, *F*(1, 93) = 1.49, *p* = .225, η²*p* = .016, was found. Nevertheless, the effect of Emotion reached significance, *F*(1, 93) = 46.09, *p* < .001, η²*p* = .345: the weighted confidence for angry-to-ambiguous (*M* = 1.76; *SE* = 0.06; 95% CI [1.65, 1.88]) was significantly larger than happy-to-ambiguous faces (*M* = 1.42; *SE* = 0.05; 95% CI [1.32, 1.52]). Null effects of SPQ were also observed upon splitting the high and low groups based on the tertile split (all *p* > .05).

When they were added individually as covariates, the SPQ (total score and factors), total PDI, total LSHS, total STICSA-1, total STICSA-2, and total TMT-A did not have significant effects (all *p* *<* .05). The main effect of Emotion remained unchanged, with the exception of a null effect using the interpersonal SPQ factor, *p* *=* .357, η²*p* = .009, and the TMT-A as covariates, *p* *=* .363, η²*p* = .009. Moreover, when using the Letter-Number Span as a covariate, results showed a main effect of memory, *F*(1, 93) = 6.79, *p* *=* .011, η²*p* = .068, and a significant Emotion by Letter-Number Span interaction, *F*(1, 93) = 7.86, *p* *=* .006, η²*p* = .078, whereas the effect of Emotion did not reach statistical significance, *F*(1, 93) = 0.90, *p* *=* .344, η²*p* = .010.

Additional analyses with Emotion and Group divided by a median split of PDI and LSHS revealed neither a main effect nor a significant interaction with Group for both PDI and LSHS (all *p* < .05). Finally, Spearman’s rank correlation showed no significant relationship between weighted confidence and total SPQ (*r* = −0.006, *p* = .952), cognitive-perceptual SPQ (*r* = −0.028, *p* = .790), interpersonal SPQ (*r* = 0.006, *p* = .957), disorganised SPQ (*r* = −0.017, *p* = .873), total LSHS (*r* = −0.108, *p* = .298), and total PDI (*r* = −0.070, *p* = .500).

### Ambiguity threshold

Slightly angrier female faces (*M* = 10.12; *SE* = 0.16; 95% CI [9.81, 10.43]) and slightly angrier male faces (*M* *=* 10.30; *SE* = 0.16; 95% CI [10.00, 10.61]) were perceived as ambiguous. The ambiguity threshold was statistically different from 11 in both female, *t*(94) = −5.68, *p* < .001, *d* = −0.583, 95% CI [−0.799, −0.364], and male faces, *t*(94) = −4.46, *p* < .001, *d* = −0.458, 95% CI [−0.668, −0.245]. There were no main effects of SPQ Group, *F*(1, 93) = 1.14, *p* = .289, η²*p* = .012, or Avatar Sex, *F*(1, 93) = 1.79, *p* = .184, η²*p* = .019, and no significant SPQ Group by Avatar Sex interaction, *F*(1, 93) = 0.14, *p* = .705, η²*p* = .002. Spearman’s rank correlation demonstrated a positive correlation between the ambiguity threshold and the cognitive-perceptual SPQ (*r* = 0.222, *p* = .030), but not total SPQ (*r* = 0.083, *p* = .422), interpersonal SPQ (*r* = 0.083, *p* = .425), disorganised SPQ (*r* = −0.127, *p* = .221), total LSHS (*r* = 0.122, *p* = .240), and total PDI (*r* = 0.157, *p* = .129).

Similar to RM analyses, JND was computed and used as a dependent variable. Descriptive analyses demonstrated that JND was greater for female faces (*M* *=* 0.94; *SE* *=* 0.5; 95% CI [0.85, 1.03]) than male faces (*M* *=* 0.89; *SE* *=* 0.05; 95% CI [0.81, 0.98]). Still, the repeated-measures ANOVA revealed no main effects of Avatar Sex, *F*(1, 93) = 0.68, *p* *=* .412, η²*p* = .007, and SPQ Group, *F*(1, 93) = 2.30 *p* *=* .132, η²*p* = .024, and no significant SPQ Group by Avatar Sex interaction, *F*(1, 93) = 0.46, *p* = .499, η²*p* = .005.

## Discussion

In this study, we aimed to explore, for the first time, emotional anticipation in response to dynamic emotional faces and its potential association with schizotypal traits, thus contributing to the understanding of dynamic emotion perception along the psychotic continuum. To achieve this, we conducted a probe judgement task with angry-to-ambiguous and happy-to-ambiguous faces, measuring PSE and JND (first-order task), alongside subjective confidence (second-order task).

### Increased anticipation of threat

We first observed a forward bias for happy-to-ambiguous faces, with the PSE being significantly larger than 11. However, such bias was not evident for angry-to-ambiguous faces, where the PSE remained statistically similar to 11. Consistently, there was a distinct pattern in the distribution of “equal” responses, characterised by a peak occurring one level beyond the endpoint for happy-to-ambiguous faces, and at the endpoint for angry-to-ambiguous faces. These findings suggest that an ambiguous face is consistently misperceived as angrier when the emotional face begins with a happy expression. As JND analyses revealed that emotion did not influence participants’ visual precision for detecting a change in facial expression, the significantly larger PSE observed in happy-to-ambiguous than angry-to-ambiguous faces may suggest that observers remained confident in their perceptual judgement despite experiencing a perceptual bias.

Although we also anticipated a significant, albeit smaller, forward bias for angry-to-ambiguous faces, our findings confirm Greenstein et al.’s (2016) study, in which moving objects signalling a presumable social threat generated greater forward displacement than neutral objects. According to the authors, this effect may facilitate a more efficient anticipation of the trajectory and/or action of a threatening stimulus. Indeed, visual and cognitive biases are consistently observed in response to (static) threatening stimuli, such as pictures of snakes and angry humans (e.g., Cole et al., 2013; Gomes et al., 2018; Leibovich et al., 2016; Öhman et al., 2012; Soares et al., 2014). These biases are thought to enhance the detection and reaction to an imminent danger (see Öhman, 2005). For instance, the perceived distance between an observer and an aggressive human is significantly smaller compared with disgusting and neutral humans (Cole et al., 2013). Hence, instead of eliciting an optimal memory representation, dynamic emotional faces with a perceptual history compatible with an impending state of anger appear to consistently trigger systematic memory distortion, leading to heightened anger anticipation. This may reflect an adaptative mechanism, as it is safer to anticipate any hostility from a conspecific than the opposite.

Besides this anger anticipation, a possible explanation could be that angrier (compared with happier) probes are more often selected, irrespective of their perceptual history. But this would likely lead to a backward shift for angry-to-ambiguous faces, which was not the case: although it did not reach statistical significance, the PSE was still larger than 11. Furthermore, if there were disparities in emotion or physical changes between angrier and happier probes, this could potentially lead to a main effect of Emotion, not directly related to RM. This scenario is improbable, given that avatar stimuli allow for more controlled and precise adjustments of anger/happiness intensity and that equidistant probes were similar in terms of pixel-wise difference between them and the animation endpoint (see Supplementary materials).

### Null effect of schizotypal traits in Representational Momentum

With respect to the effect of schizotypal traits, no significant difference emerged between the high and low SPQ groups in terms of PSE and JND. The obtained results failed to substantiate our hypothesis that individuals with high (compared with low) schizotypal traits would show a greater RM, especially concerning happy-to-ambiguous faces. Further analyses also revealed no main effect or correlation with cognitive-perceptual, interpersonal, and disorganised schizotypal traits, nor with hallucination predisposition or delusional ideation (evaluated with the LSHS and PDI, respectively). These results suggest that individuals with psychotic proneness, at a subclinical level, maintain their ability to anticipate others’ emotions, even when confronted with a likely imminent angry state. Similarly, Jarrett et al. (2002) found a comparable forward displacement among individuals with high (*N* *=* 8) and low schizotypal traits (*N* *=* 8) for non-emotional stimuli. Despite a probable lack of statistical power due to the small sample size, their findings may still reflect typical motion extrapolation patterns in schizotypy. This contrasts with Vettise’s (2012) study, which reported a positive correlation between RM and the disorganised factor of the SPQ. Vettise proposed that individuals with disorganised schizotypy may have an erratic cognitive representation of the world, potentially hindering the inhibition of automatic motion extrapolation processes. However, due to the weak correlation (*r* = 0.13), RM alone was not considered a robust marker of psychotic proneness. With this in mind, it is thus possible that schizotypal traits may not have a significant impact on motion extrapolation or anticipation processes.

Although we did not observe an effect of the SPQ group in the ambiguity threshold task for either PSE or JND, there was a positive correlation between the cognitive-perceptual SPQ factor and the ambiguity threshold. This may suggest that, when faces are presented statically, higher self-reports of positive schizotypy (e.g., suspiciousness, ideas of reference, unusual perceptual experiences) are associated with an ambiguity threshold slightly biased towards a happier expression. In other words, individuals with higher levels of positive schizotypy may tend to require more happy cues to judge a face as ambiguous. This aligns with previous descriptions of a threat-related bias for neutral faces in individuals at ultra-high risk (e.g., Seo et al., 2020; van Rijn et al., 2011) and in high schizotypal traits (e.g., Brown & Cohen, 2010), which also aligns with the hypothesis that psychotic experiences, namely persecutory delusions, are associated with an exaggerated threat perception in ambiguous events (see M. J.Green & Phillips, 2004). Importantly, the positive correlation between the cognitive-perceptual SPQ and the ambiguity threshold, alongside the null effect of schizotyp al traits in RM, could imply that positive schizotypy is linked to an anger bias for ambiguous faces solely when explicit discrimination of emotion is required and that this association does not persist when a more automatic emotion perception process is engaged. In fact, distinct meta-analyses have consistently shown impaired emotion recognition in schizophrenia (Kohler et al., 2010; Savla et al., 2013), but the evidence is mixed for paradigms less susceptible to the interference of higher-level social cognition (e.g., Caruana & Seymour, 2021; Grave et al., 2021; see Lee et al., 2016). On that note, it could be that the null effect of schizotypal traits in RM was due to participants relying less on the emotion content *per se* to make their same/different judgements, but more on the physical properties of the stimulus. Nevertheless, this seems implausible given that the RM was affected by emotion—which would not be the case if only physical properties were attended, thus strengthening the idea that our outcomes reflect intact emotional anticipation for socio-emotional events in psychotic proneness. Still, caution is needed due to the weak strength of the correlation (*r* = 0.222, *p* = .030) and to the possibility of biased results when conducting multiple correlations, as a higher number of tests increases the likelihood of finding a significant *p-*value by change alone. Indeed, despite evidence of abnormal emotion recognition in schizotypal traits (see Giakoumaki, 2016; Zouraraki et al., 2023), this effect is far from being robust, with some authors failing to find an association between facial emotion perception and schizotypal traits (e.g., Toomey & Schuldberg, 1995; van’t Wout et al., 2004; Waldeck & Miller, 2000). For instance, Rus-Calafell et al. (2013) showed no correlation between schizotypal traits and emotion recognition for both static and dynamic face stimuli.

Although statistical significance was not reached, data visualisation revealed a trend for larger PSEs in the presence of happy-to-ambiguous faces in the high versus low SPQ group. Similarly, we observed that the effect of Emotion on PSE varied depending on individual scores of delusional ideation (assessed with the PDI), and that splitting the sample into high and low delusional ideation (instead of high and low schizotypal traits) resulted in a marginally significant interaction between emotion and PDI group, with data visualisation also showing a pattern of larger PSE for happy-to-ambiguous faces in the high than low PDI group. Speculatively, the effect of psychotic-like experiences may be more extreme for individuals diagnosed with schizophrenia, perhaps leading to a significant difference between clinical and non-clinical groups. This would suggest that exaggerated emotional (or anger) anticipation occurs in the clinical manifestation of schizophrenia, but not at a subclinical level. Further studies tackling this assumption are required. In addition, De Sá Teixeira et al. (2013) demonstrated that the effect of velocity in RM—with greater RM magnitude at faster dynamics (see Hubbard, 2010, 2014)—is null in individuals with schizophrenia. Even though they used non-emotional stimuli, it may be the case that the manipulation of velocity enhances a potential effect of schizotypy. In this vein, we recommend future studies to test this hypothesis; it was not considered here because adding velocity would significantly increase the number of trials (due to the seven probe levels). In addition, caution is needed as it is also plausible that the study was underpowered and that a potential effect of psychotic-like experiences would emerge with a larger sample.

Finally, it is worth emphasising that our use of the terms “high” and “low” refers to the distribution of scores collected in this sample, which lacks the desired variability to derive stronger conclusions (*M* = 18.16; *SD* = 9.64), especially at the higher end of the scale because the maximum value obtained in our sample was 39 out of 74. This could reflect a selection bias because most of the recruited sample was composed of university students, which should be considered a limitation. Tentatively, a significant difference could emerge for individuals scoring higher on the SPQ, closer to the higher end of the psychotic continuum. A straightforward replication of this study recruiting participants with a wider range of SPQ scores emerges as a particularly relevant future direction. Also, it is possible that participants have manipulated their self-report of psychotic-like experiences to please the examiner or confirm social norms/expectations, a phenomenon known as social desirability bias (e.g., Holtgraves, 2004; Ladea et al., 2020). Although no reliable method has been developed to measure schizotypal traits, a previous study successfully used a machine learning technique to classify individuals with schizotypy based on electroencephalography data collected during an audiovisual emotion perception task (Jeong et al., 2017). Therefore, future studies could benefit from the use of more objective measures for discriminating between “high” and “low” schizotypal groups.

### Null effect of schizotypal traits in subjective confidence

Concerning the second-order task, no significant difference was found between individuals with high and low SPQ in their subjective confidence, and the Group by Emotion interaction was also not significant. Thus, our hypothesis that the high SPQ group would be overconfident, particularly for happy-to-ambiguous faces, was not supported. Despite evidence of overconfidence along the psychotic continuum (e.g., Köther et al., 2012; Moritz et al., 2014; Perez et al., 2020), this effect is not always observed (e.g., Faivre et al., 2021), particularly at a subclinical level (e.g., Dawes et al., 2021; Köther et al., 2018). For instance, Dawes et al. (2021) found only circumstantial evidence of an association between positive schizotypy and confidence, while disorganised schizotypy significantly predicted underconfidence for emotion recognition. Also, Köther et al. (2018) showed a comparable number of high-confidence emotion recognition errors in individuals with schizophrenia, first-degree relatives, and individuals with depression, while individuals with attenuated positive symptoms and healthy controls made significantly fewer high-confidence errors. This suggests that the ability to predict one’s emotion recognition performance is preserved in individuals with subclinical psychotic features, which may be the case in this study. In addition, it is worth noting that this null effect can also be explained by the narrow distribution of the SPQ scores in our sample, which prevented us from having two clearly distinct “high” and “low” groups (even after using tertile to split the sample).

Interestingly, results showed that subjective confidence for biased perceptual judgements was significantly lower for happy-to-ambiguous than angry-to-ambiguous faces, a pattern opposite to the RM results. Because ambiguous faces were systematically misperceived as angrier when preceded by happy faces, it is possible that the uncertainty of the perceptual judgement increased, which in turn lowered subjective confidence. This aligns with evidence that individuals can accurately predict their own perceptual performance (e.g., Keane et al., 2015). Gallagher et al. (2019) proposed that aftereffects driven by perceptual changes (i.e., sensory adaptation) can be measured equally using decision and confidence judgements, whereas aftereffects driven, at least in part, by non-perceptual factors (e.g., a biased pattern of responses when the input is ambiguous) result in a dissociation between decision and confidence judgements. As such, measuring subjective confidence can be particularly relevant as there is presently no consensus on the mechanisms underlying RM, with theoretical models proposing a wide range of possible explanations related to mental representations of physical properties (as the case of *momentum* or “mass in motion”, defined as the product of the object’s mass and velocity), low-level factors (mostly linked to vision, such as oculomotor behaviour, aftereffect, and perceptual adaptation), beliefs about physical systems and objects in motion (such as extrapolation and implicit knowledge), and a combination of all of these (see Hubbard, 2010). However, this question goes beyond the scope of this study.

### Additional considerations

Although the sample size was defined a priori and preregistered, the parameters concerning the low SPQ group were established through a brief pilot test with two volunteers. This has some potential issues as data from pilot tests may not fully represent the population of interest, leading to a biased or incomplete estimation. In addition, the parameters for the high SPQ group were defined by us based on the hypothesis and range of RM values obtained from the pilot test, instead of actual data from individuals with high schizotypal traits. Consequently, the difference between the SPQ groups may have been overestimated. If so, this could have resulted in an insufficient sample size and an underpowered study, especially because nine participants (8.65% of the total sample) were excluded from the analysis due to excessive noise. To enhance the reliability and robustness of our findings, with particular interest in the null effect of schizotypal traits, this study should be replicated with a larger sample size.

An effect of schizotypal traits may not have been evident due to the only small-to-medium magnitude of the forward bias observed in happy-to-ambiguous faces (*d* = 0.319 in the one sample *t*-test; and η²*p* = .048 for the main effect of Emotion), which contrasts with the effects reported in previous studies. For example, Prigent et al. (2018) showed a main effect of the facial expression of pain in PSE, with a η² of .59, thus representing a large effect. Hence, it would have been useful to test the paradigm before endorsing the schizotypy question and then adjust it as necessary, as our paradigm may have failed to induce large forward biases due to various methodological decisions. As an example, each picture along the angry-to-happy continuum encompasses a 5% shift in both anger and happiness, reflecting only a modest degree of change. Upon examining the absolute pixel-wise difference between the endpoint and each probe, it becomes apparent that the physical change is subtle, varying from an average of 0.35% (in probes that are one step angrier or happier than endpoint) to 0.97% pixel-wise difference (in probes that are three steps angrier or happier than endpoint; see Supplementary materials). Hence, although statistically significant, the forward bias in happy-to-ambiguous faces corresponds to a minor trend towards anticipating anger, potentially with limited implication beyond the laboratory setting. To enhance the significance of the forward bias, we would need to increase the gap between the endpoint and each probe while still observing an RM effect.

Another aspect worthy of consideration is the difference in duration of the first and final frames, which lasted 200 ms compared to the 80 ms of other frames. Although some authors have also opted to extend the duration of the first and/or final frame (e.g., Palumbo et al., 2015, 2018), others have maintained uniform presentation times for all frames within the animation (e.g., Prigent et al., 2018; Thornton, 2014; Uono et al., 2014; Yoshikawa & Sato, 2008). Typically, a longer presentation time allows for a more detailed processing of facial features and emotional cues, thereby enhancing emotion perception (e.g., Neath & Itier, 2014, who found improvements with presentation duration ranging from 50 to 100 ms). Although initially perceived as beneficial—particularly given that individuals with schizophrenia (and arguably individuals with psychotic proneness) may require longer stimulus exposition to accurately perceive emotional cues—we now recognise that the longer presentation time may have lessened the perceptual bias. Specifically, a more detailed processing of the final frame may have fostered a closer “match” between participants’ perceptions and the actual stimulus. This highlights the importance of considering how presentation parameters can influence perception and stresses the complexity of balancing methodological decisions to minimise unintended effects while enhancing the validity and reliability of the findings.

Finally, the subjective ambiguity did not represent the actual point of ambiguity for each stimulus, as the ambiguity threshold significantly differed from 11 for both male and female avatars. Thus, it would be beneficial to conduct a calibration before the main task to determine the perceptive ambiguity threshold for each participant and stimulus. This approach would enable us to adjust the endpoint of each animation accordingly.

## Conclusion

Ambiguous faces were found to be perceived differently depending on their immediate perceptual history. Particularly, there was an increased tendency to anticipate anger in ambiguous faces that began with happiness (happy-to-ambiguous faces), in line with a greater RM for stimuli whose dynamics are congruent with an imminent threat. Nevertheless, our preregistered hypothesis that this effect would be stronger in individuals with high schizotypal traits was not supported, thus suggesting that emotional anticipation might not be a potential marker of psychotic proneness. Finally, we also did not observe a significant overconfidence in biased perceptual judgements by individuals with high schizotypal traits. Still, caution is essential when interpreting these findings, as a hypothetical effect of schizotypal traits or other psychotic-like experiences may have been reduced due to the use of self-report questionnaires, recruitment of university students, and/or a too-small sample.

Altogether, the results showed the usefulness and promising value of RM as a tool to explore the perception of socio-emotional dynamic stimuli, mirroring the relevance of dynamic information and time-unfolding events in social interactions, while opening research avenues concerning emotional anticipation in the clinical manifestation of schizophrenia.

## Supplemental Material

sj-docx-1-qjp-10.1177_17470218241253703 – Supplemental material for Emotional anticipation for dynamic emotional faces is not modulated by schizotypal traits: A Representational Momentum study Supplemental material, sj-docx-1-qjp-10.1177_17470218241253703 for Emotional anticipation for dynamic emotional faces is not modulated by schizotypal traits: A Representational Momentum study by Joana Grave, Sara Cordeiro, Nuno de Sá Teixeira, Sebastian Korb and Sandra Cristina Soares in Quarterly Journal of Experimental Psychology

## Notes

1. Anti-facial expressions are facial expressions in which the facial feature displacement is similar in magnitude, but opposite in direction, in relation to their corresponding normal facial expression (Uono et al., 2021).

2. For the low SPQ group, happy-to-ambiguous and angry-to-ambiguous means were defined based on a pilot testing with two volunteers. Then, those values were considered to estimate the means for the high SPQ group, assuming that people with high schizotypal traits are more prone to perceive anger overall, especially in faces transitioning from happiness to ambiguity. For a discussion on potential issues associated with this approach, please refer to Section 4.4.

3. In the preregistration, the dependent variable consisted of a weighted mean RM, in which the sum of the product of the proportion of “equal” responses and the probe level was divided by the sum of the proportion of “equal” responses. Despite its common usage in the RM literature (e.g., Munger, 2015; Thornton, 2014), we found that this method did not effectively identify outliers in our study: the weighted mean necessarily falls within the range of −3 to 3 (representing the “distance” between the endpoint and the angriest or happiest probes), which could inadvertently hide issues within the data, such as noise or random distribution of “equal” responses. For instance, in the case of one participant (ID93), the weighted mean was −0.54 for happy-to-ambiguous faces and −0.21 for angry-to-ambiguous faces. Although these values may appear reasonable, the response distribution did not follow a consistent pattern, with the proportion of “equal” responses varying widely across different probe levels: 0.67, 0.67, 0.17, 0.58, 0.33, 0.50, and 0.17 for probe levels 8, 9, 10, 11, 12, 13, and 14, respectively. Taking these considerations into account and in alignment with recommendations provided during peer review, we chose to use PSE as a dependent variable. This approach enabled us to identify potential outliers and obtain a more robust estimate of the memory bias.

4. Despite subjective confidence ranging between 1 and 4, the descriptive analysis showed little variation within our sample (*M* = 3.00, *SD* = 0.39, Min *=* 2.05, Max *=* 3.94). Therefore, the preregistered analysis was not feasible, as it would reflect the outcomes obtained in RM. Weighted confidence mean was used instead.
