# Supplementary material for: Emotional anticipation for dynamic emotional faces is not modulated by schizotypal traits: A Representational Momentum study
Source: Q J Exp Psychol (Hove). 2024 May 16;78(6):1088–106. doi: 10.1177/17470218241253703 (PMC12095887; doi:10.1177/17470218241253703)
Supplement: sj-docx-1-qjp-10.1177_17470218241253703 – Supplemental material for Emotional anticipation for dynamic emotional faces is not modulated by schizotypal traits: A Representational Momentum study [file sj-docx-1-qjp-10.1177_17470218241253703.docx]

Supplementary Material for:

**Emotional anticipation for dynamic emotional faces is not modulated by schizotypal traits: A Representational Momentum study**

Joana Grave^a,b*^, Sara Cordeiro^c^, Nuno de Sá Teixeira^a^, Sebastian Korb^d,e^, Sandra C. Soares^a*^

^a^William James Center for Research (WJCR-Aveiro), Department of Education and Psychology, University of Aveiro, Campus Universitário de Santiago, 3810-193 Aveiro, Portugal

^b^Center for Health Technology and Services Research (CINTESIS@RISE), Department of Education and Psychology, University of Aveiro, Campus Universitário de Santiago, 3810-193 Aveiro, Portugal

^c^Department of Education and Psychology, University of Aveiro, Campus Universitário de Santiago, 3810-193 Aveiro, Portugal

^d^Department of Psychology, University of Essex, CO4 3SQ Colchester, United Kingdom

^e^Department of Cognition, Emotion, and Methods in Psychology, University of Vienna, Liebiggasse 5 1010, Vienna, Austria

*Correspondence should be addressed to Joana Grave ([joanagrave@ua.pt](mailto:joanagrave@ua.pt)) and Sandra C. Soares ([sandra.soares@ua.pt](mailto:Sandra.soares@ua.pt)), Department of Education and Psychology, University of Aveiro, Campus Universitário de Santiago, 3810-193 Aveiro, Portugal.

**Supplementary Materials**

**Participants**

**Table S1.** Sociodemographic, psychological, and cognitive characterization of the sample.

|  |  | Total  (*N* = 95) | High SPQ  (*N* = 46) | Low SPQ  (*N* = 49) |
| --- | --- | --- | --- | --- |
| Age, *M* (*SD*) | | 21.51 (4.23) | 20.63 (3.91) | 22.33 (4.40) |
| Sex, *N* (*%*) | Female | 67 (70.53) | 34 (73.91) | 33 (67.34) |
|  | Male | 28 (29.47) | 12 (26.09) | 16 (32.65) |
| Nationality, *N* (*%*) | Portuguese | 88 (92.63) | 41 (89.13) | 47 (95.92) |
|  | Brazilian | 6 (6.31) | 4 (8.70) | 2 (4.08) |
|  | Other | 1 (1.05) | 1 (2.17) | 0 (0.00) |
| Education, *N* (*%*) | ≤ 12 years | 58 (61.05) | 35 (76.09) | 24 (49.00) |
|  | Graduation | 25 (26.31) | 7 (15.21) | 17 (34.69) |
|  | Master | 10 (10.53) | 4 (8.70) | 6 (12.25) |
|  | ≥ PhD | 2 (2.11) | 0 (0.00) | 2 (4.08) |
| SPQ, *M* (*SD*) | Total score | 18.16 (9.64) | 26.61 (5.52) | 10.22 (4.54) |
|  | Disorganized | 3.26 (2.61) | 4.78 (2.39) | 1.84 (1.92) |
|  | Interpersonal | 10.80 (5.68) | 15.30 (4.16) | 6.57 (3.08) |
|  | Cognitive-perceptual | 6.42 (5.25) | 9.91 (5.10) | 3.14 (2.64) |
| PDI-21, *M* (*SD*) |  | 4.03 (3.06) | 5.52 (3.27) | 2.63 (2.04) |
| LSHS-R, *M* (*SD*) |  | 14.71 (10.13) | 21.57 (10.80) | 8.25 (5.59) |
| STICSA-1, *M* (*SD*) | | 30.03 (6.69) | 32.54 (7.21) | 27.67 (5.21) |
| STICSA-2, *M* (*SD*) | | 32.39 (8.35) | 36.61 (8.39) | 28.43 (6.12) |
| TMT-A, *M* (*SD*) | | 30.04 (9.68) | 30.34 (10.37) | 29.76 (9.08) |
| Letter-number Span | | 10.89 (2.22) | 11.02 (1.93) | 10.78 (2.48) |

*Notes.* LSHS-R, Launay-Slade Hallucination Scale-Revision; SPQ, Schizotypal Personality Questionnaire; STICSA-1; Inventory for Cognitive and Somatic Anxiety-State, STICSA-2; Inventory for Cognitive and Somatic Anxiety-Trait; PDI-21, Peters et al. Delusional Inventory; TMT-A, Trial Making Test – Part A.

**Psychological Assessment**

*Launay-Slade Hallucination Scale-Revision*

Besides schizotypy, we measured psychotic-like experiences that can occur at a subclinical level, thus allowing a more detailed assessment of psychosis-proneness. The Portuguese version of the Launay-Slade Hallucination Scale-Revision (LSHS-R; Castiajo & Pinheiro, 2017) was used to evaluate different forms of hallucinations, namely auditory, visual, olfactory, tactile, hypnagogic, and hypnopompic. This self-report scale, originally developed by Launay and Slade (1981), contains 16 items measured with 5-point Likert scales from 0 (“definitely does not apply to me”) to 4 (“definitely applies to me”). Total score ranges from 0 to 64, with higher scores indicating higher hallucination predisposition. In our sample, Cronbach’s Alpha for the total score was 0.88, also suggesting good internal consistency.

*Peters et al. Delusional Inventory*

We used the Portuguese version of the Peters et al. Delusional Inventory (PDI-21; Pimentel et al., 2017), originally developed by Peters et al., (2004), to evaluate the multidimensionality of delusional ideation. This self-report inventory includes 21 items with a dichotomous response format (yes/no). Each “yes” response is scored 1. For each “yes” response, participants are asked to rate the degree of distress, preoccupation, and conviction using 5-point Likert scales. Total score ranges from 0 to 21, with higher scores indicating higher delusional ideation. It is also possible to compute scores for distress, preoccupation, and conviction, by summing up the “yes” responses in each dimension (“no” responses are automatically scored 0). Cronbach’s Alpha in our sample was 0.76, suggesting acceptable internal consistency.

*State-Trait Inventory for Cognitive and Somatic Anxiety*

To control for the effects of anxiety, we used the Portuguese version of the State-Trait Inventory for Cognitive and Somatic Anxiety (STICSA; Barros et al., 2022). The STICSA, originally developed by Ree et al. (2008), contains two subscales of 21 items each: a state-anxiety scale (STICSA-1), which asks for the evaluation of how the individual “feels at this moment”; and a trait-anxiety scale (STICSA-2), referring to “how often, in general, the sentence is true for you”. All items are measured in a 4-point frequency response scale, ranging from 1 (“nothing”) to 4 (“very”). Total score varies between 21 and 84, with higher scores indicating higher anxiety. In our sample, we found a Cronbach’s alpha of 0.85 for STICSA-1 and 0.90 for STICSA-2, indicating good internal consistency.

*Cognitive Assessment*

We used the Portuguese version of the Trail Making Test Part A (TMT-A; Cavaco et al., 2013) and the Letter-Number Span (Wechsler, 2008) to control for the effects of cognition. These instruments were selected because they are part of the MATRICS Consensus Cognitive Battery, a standardized battery to measure cognitive domains relevant to schizophrenia and related conditions (Nuechterlein et al., 2008).

The TMT-A measures attention, visual scanning, and information processing speed. In this test, participants are asked to connect sequentially encircled numbers (from 1 to 25) on a page, as quickly and accurately as possible. The examiner gives feedback whenever an error is made, and the test is discontinued after 200 sec or 4 errors (unless the participant is less than 3 numbers from the end). In the present study, we measured the time to complete the test (in sec). Shorter time of completion indicates better performance.

The Letter-Number Span evaluates verbal working memory. In this test, the examiner verbally presents a set of intermixed letters and digits that increase by one on each trial, starting from the length of two up to maximum of eight stimuli. Each trial contains three sequences of the same length, resulting in a total of 21 stimuli distributed by seven trials. Participants are asked to memorize the stimuli in each sequence and to repeat the digits in ascending order, followed by the letters in alphabetic order. The test is discontinued after three errors in consecutive sequences within the same trial. Total score is the number of correctly recalled sequences, with a maximum score of 21. Higher scores indicate better working memory performance.

**Stimuli**

We used the *package* imager (Barthelme, 2023) in RStudio (RStudio Team, 2022) to calculate the absolute pixel-wise difference between the final frame of the inducing sequence and each of the seven frames used as probes (Table S2). Given that each frame encompasses 800 × 1200 pixels, a maximum absolute difference of 10207.05 (in a female avatar with 65% anger and 35% happiness) corresponds to a 1.06% change, whereas the minimum difference observed was 2963.78 (in a male avatar with 45% anger and 55% happiness), representing a 0.31% change. See Fig. S1 for a visualization of the absolute pixel-wise difference in a male avatar.

**Table S2.** Absolute pixel-wise difference between the final frame of the dynamic sequence and each of the seven possible probes.

|  |  | AH_07 | AH_08 | AH_09 | AH_10 | AH_11 | AH_12 | AH_13 |
| --- | --- | --- | --- | --- | --- | --- | --- | --- |
| Avatar | M1 | 9374.79 | 6532.48 | 3410.67 | 0 | 3371.14 | 6536.78 | 9413.69 |
|  | M4 | 8302.92 | 5752.58 | 3008.14 | 0 | 2963.78 | 5744.39 | 8304.72 |
|  | F1 | 10207.05 | 7091.85 | 3686.87 | 0 | 3602.44 | 6931.66 | 9902.98 |
|  | F4 | 9430.29 | 6549.17 | 3413.69 | 0 | 3339.95 | 6431.07 | 9234.16 |

*Notes.* AH_07: 65% anger and 35% happiness; AH_08: 60% anger and 40% happiness; AH_09: 55% anger and 45% happiness; AH_10: 50% anger and 50% happiness, corresponding to the final frame; AH_11: 45% anger and 55% happiness; AH_12: 40% anger and 60% happiness; AH_13: 35% anger and 65% happiness.


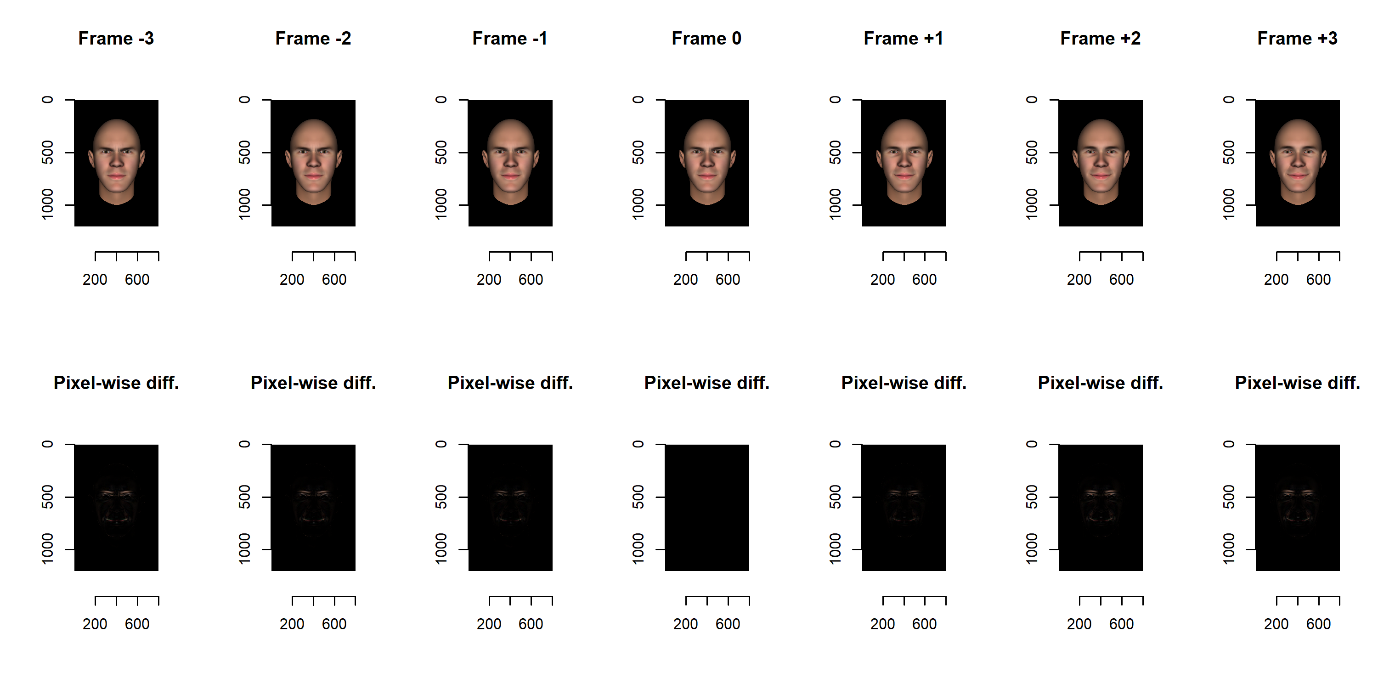


**Fig. S1.** Visualization of the absolute pixel-wise difference in an angry-to-ambiguous male face.

To confirm the similarity in absolute pixel-wise difference values of two analogous probes – those equidistant from the endpoint –, we ran separated Wilcoxon Signed Rank Tests using the *wilcox.test* function. As anticipated, the tests revealed a non-significant difference between 3-step probes, *V* = 7, *p* = .625, 2-step probes, *V* = 9, *p* = .250, and 1-step probes, *V* = 10, *p* = .125.

**References**

Barros, F., Figueiredo, C., Brás, S., Carvalho, J. M., & Soares, S. C. (2022). Multidimensional assessment of anxiety through the State-Trait Inventory for Cognitive and Somatic Anxiety (STICSA): From dimensionality to response prediction across emotional contexts. *PLOS ONE, 17*(1), e0262960. <https://doi.org/10.1371/journal.pone.0262960>

Barthelme, S. (2023). *_imager: Image processing library based on ‘CImg’_* [*R package version 0.45.2]* [Computer software]. <https://CRAN.R-project.org/package=imager>

Castiajo, P., & Pinheiro, A. P. (2017). On ‘hearing’ voices and ‘seeing’ things: Probing hallucination predisposition in a Portuguese nonclinical sample with the Launay-Slade Hallucination Scale-Revised. *Frontiers in Psychology, 8*, 1138. <https://doi.org/10.3389/fpsyg.2017.01138>

Cavaco, S., Gonçalves, A., Pinto, C., Almeida, E., Gomes, F., Moreira, I., Fernandes, J., & Teixeira-Pinto, A. (2013). Trail Making Test: Regression-based Norms for the Portuguese Population. *Archives of Clinical Neuropsychology, 28*(2), 189–198. <https://doi.org/10.1093/arclin/acs115>

Launay, G., Slade, P. D. (1981). The measurement of hallucinatory predisposition in male and female prisoners. *Personality and Individual Differences, 2*(3), 221–234. <https://doi.org/10.1016/0191-8869(81)90027-1>

Nuechterlein, K. H., Green, M. F., Kern, R. S., Baade, L. E., Barch, D. M., Cohen, J. D., Essock, S., Fenton, W. S., Frese, F. J., Gold, J. M., Goldberg, T., Heaton, R. K., Keefe, R. S. E., Kraemer, H., Mesholam-Gately, R., Seidman, L. J., Stover, E., Weinberger, D. R., Young, A. S., … Marder, S. R. (2008). The MATRICS Consensus Cognitive Battery, part 1: Test selection, reliability, and validity. *The American Journal of Psychiatry, 165*(2), 203–213. <https://doi.org/10.1176/appi.ajp.2007.07010042>

Peters, E., Joseph, S., Day, S., Garety, P. (2004). Measuring delusional ideation: The 21-items Peters et al. Delusions Inventory (PDI). *Schizophrenia Bulletin, 30*(4), 1005-1022. <https://doi.org/10.1093/oxfordjournals.schbul.a007116>

Pimentel, S., Cunha, M., Galhardo, A., & Couto, M. (2017). Validation of the delusional ideation inventory for the Portuguese population. *European Psychiatry, 41*, S809. <https://doi.org/10.1016/j.eurpsy.2017.01.1566>

Ree, M. J., French, D., Macleod, C., Locke, V. (2008). Distinguishing cognitive and somatic dimensions of state and trait anxiety: Development and validation of state-trait inventory for cognitive and somatic anxiety (STICSA). *Behavioural and Cognitive Psychotherapy, 36*(3), 313-332. [https://doi.org/10.1017/S1352465808004232](https://psycnet.apa.org/doi/10.1017/S1352465808004232)

RStudio Team. (2022). *RStudio: Integrated developmental environment for R [version 2022.7.1.554]* [Computer software]. RStudio, PBC. <http://www.rstudio.com/>

Wechsler, D. (2008). *WAIS-III: Manual da Escala de Inteligência de Wechsler para Adultos* (3rd ed.). CEGOC-TEA [Hogrefe].
